# Minimally Invasive Laminate Veneers: Clinical Aspects in Treatment Planning and Cementation Procedures

**DOI:** 10.1155/2016/1839793

**Published:** 2016-12-13

**Authors:** R. K. Morita, M. F. Hayashida, Y. M. Pupo, G. Berger, R. D. Reggiani, E. A. G. Betiol

**Affiliations:** ^1^Graduate Pontifical Catholic University of Parana, Curitiba, PR, Brazil; ^2^Department of Dentistry, School of Dentistry, University Tuiuti of Parana, Curitiba, PR, Brazil; ^3^Department of Restorative Dentistry, Federal University of Parana, Curitiba, PR, Brazil; ^4^Graduate Federal University of Parana, Curitiba, PR, Brazil

## Abstract

When a definitive aesthetic treatment is determined, it is crucial to grant the patient's wish with the necessary dental treatment. Thus, conservative treatments that are the solution to aesthetic problems involving morphologic modifications and provide the result that the patient expects should always be the first therapeutic option. In this context, ceramic laminate veneers, also known as “contact lens,” are capable of providing an extremely faithful reproduction of the natural teeth with great color stability and periodontal biocompatibility. Minimal or no preparation veneers are heavily advertised as the answer to our patients' cosmetic needs, which they can be if they are used correctly in the appropriate case. This report is about ultraconservative restorations to achieve functional and aesthetic rehabilitation through treatment planning. Thus, clinicians should be aware that the preparation for laminate veneers remains within enamel, to ensure the bond strength and avoid or minimize the occurrence of postoperative sensitivity.

## 1. Introduction

One of patients' greatest desires when seeking dental treatment is the aesthetic transformation of their smiles to include healthy and harmonious dentition. Because of this, conservative treatments that are able to modify the shape, size, and color of the teeth and that provide the result that the patient expects should always be the first therapeutic option [1–3].

Contrary to what many clinicians think, the concept of ceramic laminates without tooth surface wear is not new. Historically, during the 1930s, a California dentist Charles Leland Pincus [4] worked in the US film industry; he had the difficult and privileged task of aesthetically improving the smiles of stars such as Shirley Temple, Bob Hope, Montgomery Clift, Elizabeth Taylor, Barbara Stanwyck, Fred Astaire, James Dean, Walt Disney, Judy Garland, and many others. Pincus used thin ceramic veneers with an adhesive aid for the temporary fixation of full dentures. However, due to a lack of appropriate cement, the procedure lasted only a few hours.

During the 1980s, after the development of techniques for adhesive cementation, ultrathin laminates were relaunched. However, at the time, the practice did not spread as quickly as expected, due mainly to professionals' fears regarding the strength of the very thin porcelain veneers in resisting masticatory forces [5]. Due to increasing aesthetic demand and the possibility of joining laminated ceramic to the tooth structure (particularly enamel), a new concept was introduced: minimally invasive restorative dentistry, which causes little damage to dental structures [2].

In this context, laminate veneer, also known as contact lenses, emerged. This extremely aesthetic solution uses nothing more than thin ceramic fragments but presents excellent optical properties. It is considered one of the most conservative treatments for oral rehabilitation, as it requires minimal or no tooth preparation [6, 7]. With thicknesses ranging from 0.2 to 0.5 mm, ceramic laminate is capable of providing an extremely faithful reproduction of the natural teeth with great color stability [5]. Laminate veneer also offers biocompatibility with the periodontal and dental substrates [8, 9], and it may be used with minimal wear or even without preparation [10].

Patients undergoing this type of treatment require that the ceramic laminates offer clinical longevity. However, planning is a crucial step in successful aesthetic rehabilitation treatment. Planning each clinical case using a photographic protocol provides better predictability in the final outcome. In addition to photos and videos, wax-up/mock-up binomial important tools to establish correct values and to ensure the symmetry and proportion to the new smile [2, 11, 12]. Therefore, the purpose is to describe two clinical cases in a step-by-step process from the planning through the cementing phase, showing that it is possible for ultraconservative restorations to combine planning with technical and multidisciplinary treatment.

## 2. Case Presentations

**Case 1 .**

A female patient (23 years old) with high aesthetic requirements sought dental care in private practice, complaining about the color and size of her anterior teeth because talking does not reveal her upper incisors.

**Case 2 .**

A male patient (25 years old) sought dental care in private practice due to a fracture in his maxillary central incisors after hitting the bottom of a pool while diving.

## 3. Treatment Planning

**Case 1 .**

**Figure 1:**
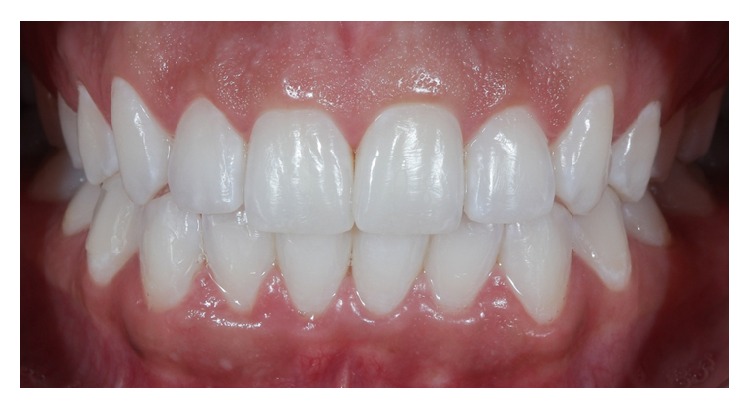
Intraoral view of the anterior maxillary teeth (Case 1).

An intraoral clinical examination revealed that the anterior teeth had a small axial inclination and asymmetric gingival margins (Figure 1), and the patient elected to plan for the removal and refurbishment of the gingival tissues through periodontal plastic surgery techniques such as gingivoplasty or gingivectomy after first fabricating ceramic laminates.

In clinical practice, especially in prosthetic restorative procedures, there is often a need for prior corrections of the contours and the gingival anatomy due to the goals of achieving facial aesthetics and ensuring harmony between the gum tissue and the dental anatomy.

**Case 2 .**

**Figure 2:**
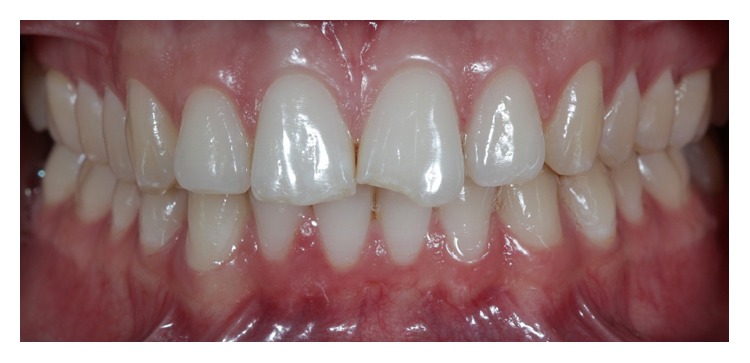
Intraoral view of the anterior maxillary teeth (Case 2).

An intraoral clinical examination revealed Class IV fracture in tooth 21 and small fractures in the incisal edge of tooth 11. The patient also presented with a diastema between the canines and the maxillary lateral incisors (Figure 2). A dental diastema is a space (or lack of contact) between two or more adjacent teeth. Diastemata are more frequent in the anterior maxilla, although they can occur in other regions of the mouth.

Regarding minimally invasive restorations, planning should not be restricted to the steps of forming and cementing. Other essential steps in the planning process for laminated ceramics include photographic and video analysis, Digital Smile Design (DSD), and mock-ups.

## 4. Photographic Protocol and Digital Planning

**Figure 3:**
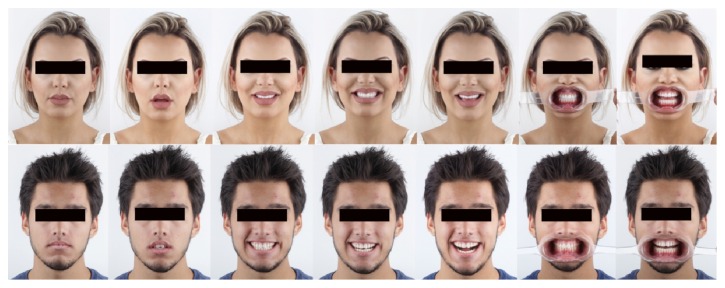
Photographic protocol for an aesthetic design and analysis of the patient's smile disharmonies.

The photographic protocols were performed (Figure 3) to assist in the evaluation of various parameters that can influence the final result, such as smile height, amplitude of the buccal corridor, lip position, dental midline, and individual characteristics of each tooth.

**Figure 4:**
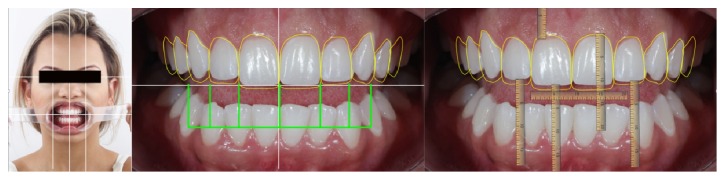
Digital Smile Design (DSD) protocol, Case 1. In this protocol, the ideal horizontal plane and vertical midline on the facial photograph were determined. After that, the cross to the intraoral photography to establish the vertical midline and occlusal plane was transferred. Furthermore, measurements of the distance were between the horizontal line and incisal edge on the photograph. Final teeth outline and gingival contour demonstrated the relationship between the preoperative situation and the final design.

**Figure 5:**
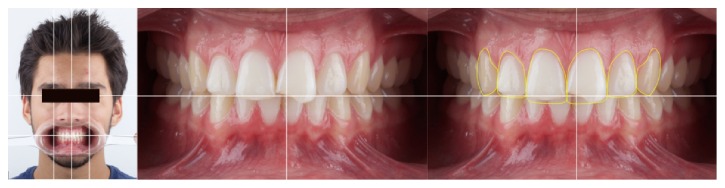
Digital Smile Design (DSD) protocol, Case 2.

With a photographic protocol, it was feasible to plan both cases using DSD. This process involves adding lines and digital designs to face photos in a specific sequence to better assess the aesthetic relationship between teeth, gums, smile, and face; this allows for a better understanding of the problems and can lead to possible solutions (Figures 4 and 5). For this study, the images were displayed on a computer using the Keynote slide show program (Apple, Cupertino, California, US), which allowed the patient to view the photos and participate in the planning. The virtual planning tool also helped in the making of the diagnostic wax models.

## 5. Diagnostic Wax-Up

**Figure 6:**
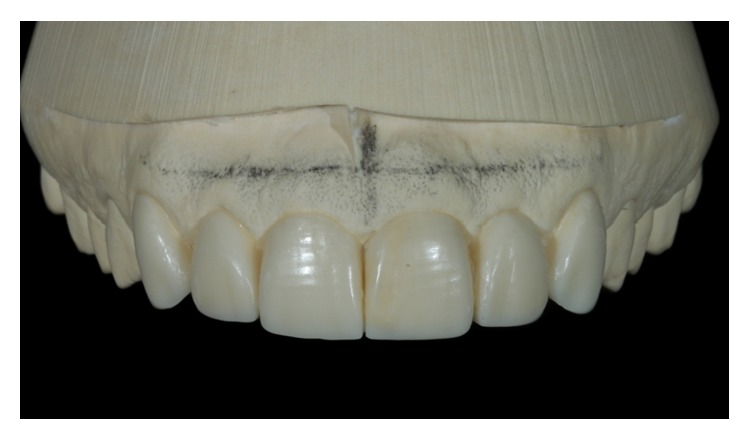
Diagnostic wax-up, Case 1.

**Figure 7:**
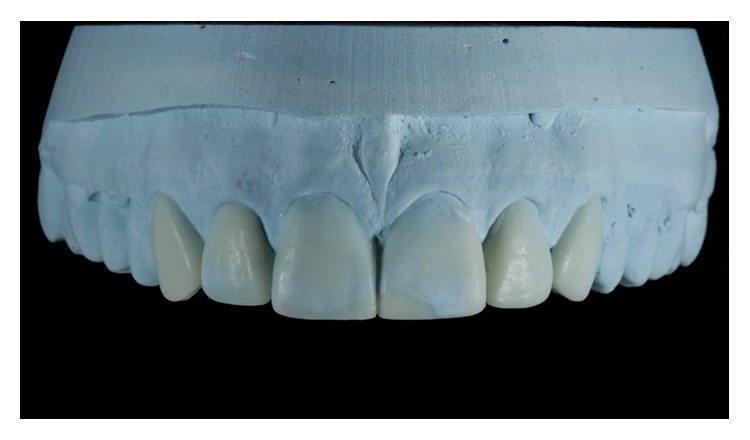
Diagnostic wax-up, Case 2.

For the case study, DSD was used to create a diagnostic wax additive that shows the individual anatomical features of the teeth. Case 1 involved an increase in mesiodistal dimensions, the cervical alignment of the incisor edges, and buccal volumes of teeth 13–23 (Figure 6). Case 2 involved the reanatomization of fractured teeth and the closing of diastema (Figure 7). After defining this process and getting the patients' approval, we conducted a test (mock-up) by placing wax directly over the patients' teeth without the need to erode the surfaces. In a mock-up, the patient can correct any of his/her dislikes [13].

**Figure 8:**
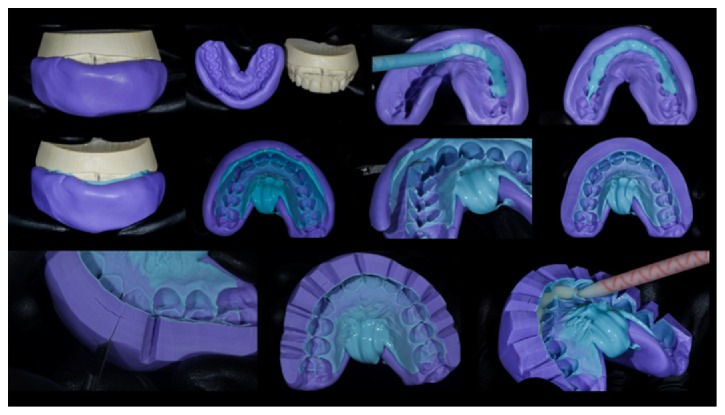
Step-by-step process for obtaining a matrix of addition silicone to make the mock-up and load it with the bis-acryl resin.

**Figure 9:**
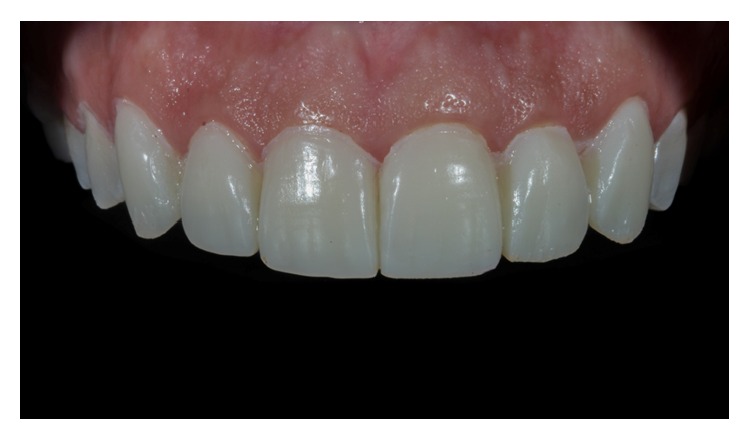
Mock-up without tooth preparation.

**Figure 10:**
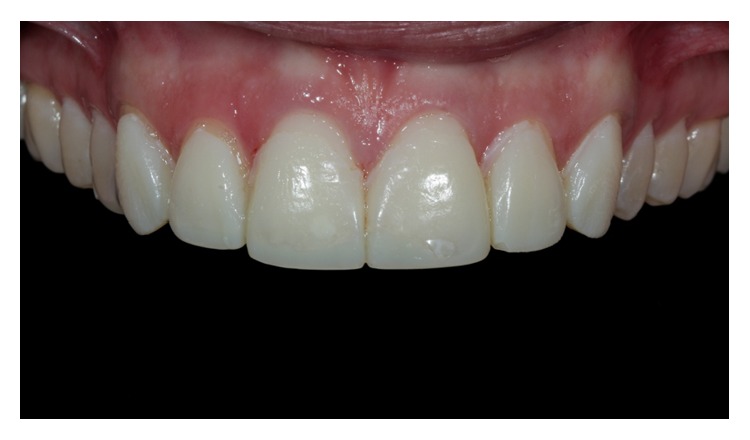
Mock-up after finishing with a scalpel blade.

Once the wax-up was completed, a silicone guide (Futura AD, DFL, Jacarepaguá, RJ, Brazil) was made and put under 2 atm of pressure to increase detail reproduction. This silicone guide was partially filled with a bis-acryl resin material (Protemp 4, 3M ESPE, St. Paul, MN, US) and placed in the patient's mouth (Figure 8). Before complete setting, a scalpel blade was used to define the correct gingival contour, respecting the manufacturer's recommendations. Gauze moistened with alcohol was used to create a polished and shiny appearance. In this way, it was possible to preview the end result of treatment (Figures 9 and 10).

## 6. Clinical and Laboratory Stages

### 6.1. Gingivoplasty

In Case 1, after the diagnostic wax-up was fabricated, gingivoplasty was performed on the predetermined elements. A period of 45 days was expected for perfect healing and maturation of the gingival tissue. In this treatment, multidisciplinary dentistry, represented by the relationship between periodontics and prosthodontics, is not restricted only to the need to observe periodontal health for the completion of a restorative treatment; it also extends to the indication of gingivoplasty to improve the treatment's final result.

### 6.2. Color Choice

The color selection was made, while the teeth were hydrated at the beginning of the consultation. Furthermore, a mapping of the colors was conducted to facilitate communication with the dental laboratory.

### 6.3. Minimally Invasive Preparations

In the case of the ultrathin ceramic restorations for the diastema closure, the insertion axis will begin in the cervical incisal direction, involving the mesial and distal surfaces [14]. Therefore, it is necessary to note areas of retention in the teeth before the molding step.

**Figure 11:**
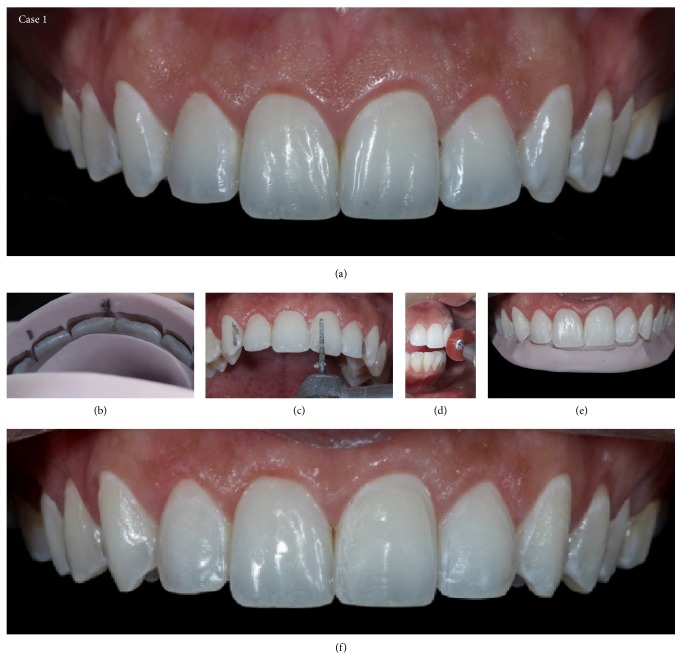
Case 1. (a) Initial view of the teeth before preparation. During treatment, the patient chose to extend the treatment to the bilateral second premolars. (b) Vestibular guide to assess the distance between the dental tissue and the correct placement indicated by the wax model, with information necessary to direct the professional in the decision of whether to wear down the sound tooth structure. (c) Palatal guide to aid the professional in determining the proper cervicoincisal size and the correct positioning of the incisal edge. (d)-(e) Minimally invasive preparations using a sanding disc and diamond burs. (f) Appearance immediately after minimally invasive preparation.

**Figure 12:**
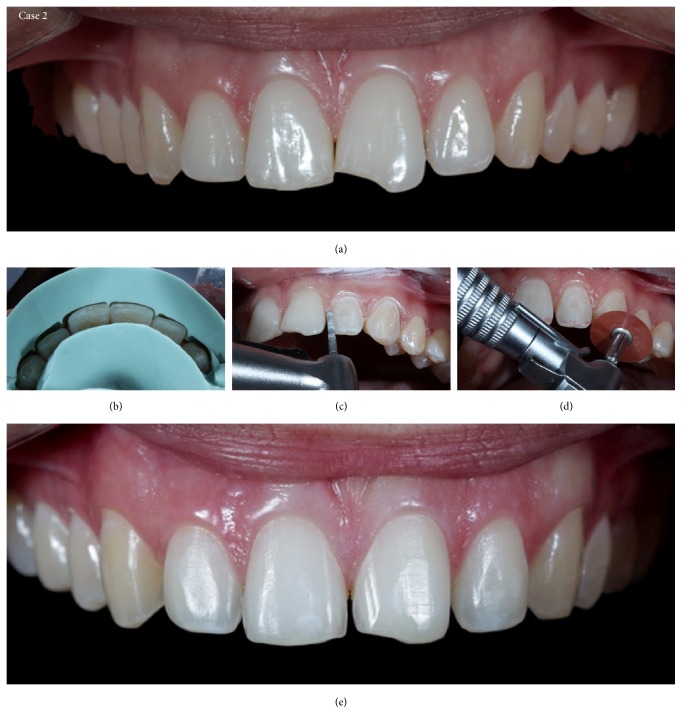
Case 2. (a) Initial view of the teeth before tooth preparation. (b) Enamel planning and demarcation of the end line of the future ceramic restoration with diamond burs. (c) Entrance exam guide to evaluate the distance between the dental tissue and the correct placement indicated by the wax model. (d) Removal of the retentive and incisal settlement areas with sandpaper discs. (e) Appearance immediately after minimally invasive preparation.

Minimally invasive preparations were performed using diamond burs and sanding discs, which were oriented with silicone guides (Zetalabor [Zhermack, Badia Polesine, Italy] and Coltène Speedex Putty [Coltène AG, Altstätten, Switzerland]) (Figures 11 and 12). During this phase, it was important to use the silicone matrix, obtained from the wax-up, to guide the amount of reduction in tooth preparation. This matrix may be constructed on the diagnostic wax study model or on the plaster model (which has provisional veneers). In these cases, the provisional veneers serve the same function as the dental wax.

### 6.4. Impression Procedure

In Case 1, the impression was carried out in two steps, with heavy and light addition silicone (Elite HD+, Zhermack) to obtain an accurate copy of the entire tooth and the gingival structures. Gingival retraction cord was used, as the terms of the preparations were all supragingival. In the first step, the dense addition silicone (Elite HD+, Zhermack) was manipulated and placed on the previously selected tray. It was placed in the patient's mouth, and internal relief was carried out simultaneous with the movements of the “strip and set.” The second step involved the use of a small amount of the same silicone rubber that was applied to the dental preparations and the first mold. The tray assembly and the dense silicone portion were placed into position on the light mixture and allowed to set.

In Case 2, the impression was performed in two steps with heavy and light bases of addition silicone (Virtual, Ivoclar Vivadent, Schaan, Liechtenstein) to obtain an accurate copy of the entire tooth and the gingival structure, following the same sequence as in Case 1. The retractor cord was not used, as the preparations were strictly supragingival. The lower jaw of each patient was molded with the same material that was used to form the antagonist of the upper jaw in the production model.

**Figure 13:**
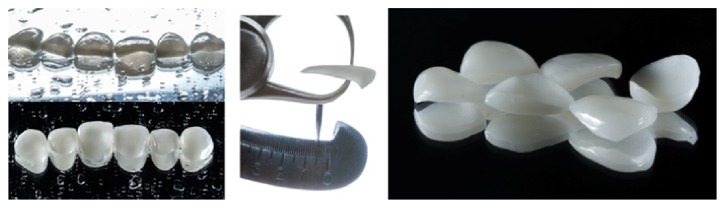
Ceramic laminate veneers.

The feldspathic porcelain laminate veneers were manufactured with IPS d.SIGN (Ivoclar Vivadent) to maintain the teeth's naturalness (Figure 13).

## 7. Try-In/Shade Selection

**Figure 14:**
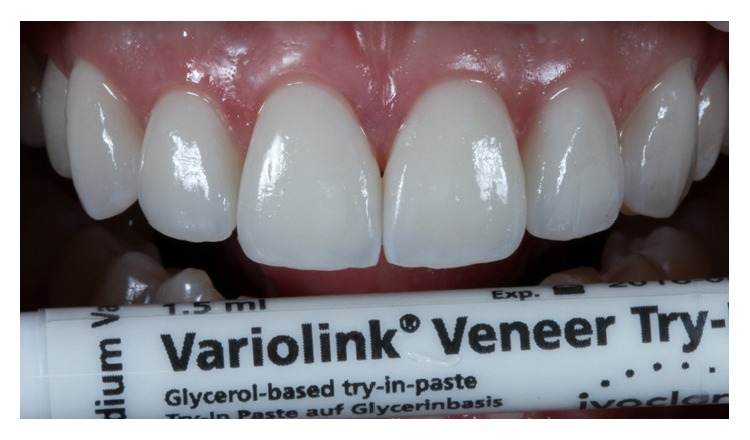
Case 2. Proofing of ceramic laminates with try-in paste (MV0, Variolink Veneer, Ivoclar Vivadent).

To minimize the chances of errors during the cementation stage, such as possible fractures in the ceramics, two types of tests were done in the mouth: dry proof and damp proof using try-in paste (Case 1: Allcem Veneer with try-in paste, [FGM, Joinville, SC, Brazil]; Case 2: Variolink Veneer with try-in paste, Ivoclar Vivadent) (Figure 14). The veneers were tested on the preparations with the help of the try-in paste in the colors Trans (Case 1) and MV 0 (Case 2), which was deposited on the inner surfaces of all the veneers. The nonpolymerizable try-in paste is soluble in water and mimics the colors of resin cement after being light-cured, providing the professional with more confidence to carry out works with great aesthetic requirements. After removing the excess paste, a snapshot was taken to evaluate the color. At that time, the patient viewed and commented on the final color of the dental elements. Therefore, 30-second application of 37% phosphoric acid is used only for cleaning, not for etching, and rinse with water and dry.

## 8. Cementation of Veneers

The cementation of the ceramic laminates is fundamental, as it will be the last step in the work; it should be done with extreme caution. It is important to remember that, unlike conventional crowns, which use dual-type resin cements, ceramic laminates should use a purely light-cured luting agent to prevent the color shifts that can occur due to chemical changes in the curing process. Furthermore, due to thin restorations such as contact lenses, which most allow photoactivation through them, there is no guarantee that the resin cement will be effectively cured.

For minimally invasive restorations, the choice was acid-sensitive ceramics, those that experience surface changes when conditioned with 10% hydrofluoric acid, because this process increases the adhesion between the restoration and the tooth.

The essential difference in the internal etching processes of ceramics is the duration of hydrofluoric acid exposure [15]. The internal surface of the restoration was etched with 9% hydrofluoric acid (Ultradent Porcelain Etch, South Jordan, UT, USA) for 90 s (etching time for feldspathic glass ceramic) [16], washed under running water, and air-dried. Insoluble silica-fluoride salts as by-products precipitate on the surface were removed by cleaning the restorations by etching and rubbing the surface with 37% phosphoric acid (Nova DFL Industry and Trade SA, Rio de Janeiro, Brazil) for 1 minute before application of the silane [17, 18]. Silane coupling agent was applied (RelyX Ceramic Primer, 3M ESPE) for 1 minute, followed by a layer of adhesive (Case 1: Ambar, FGM; Case 2: Excite, Ivoclar Vivadent), and a gently air-dried. The adhesive should not be polymerized in this stage.

**Figure 15:**
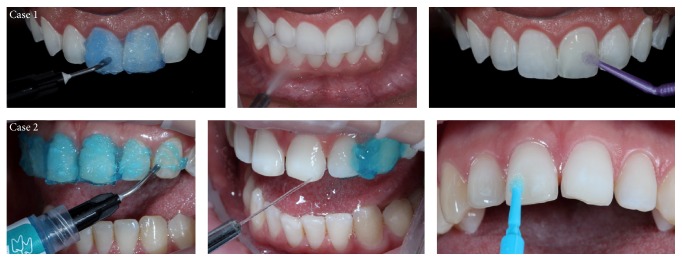
Etching with phosphoric acid at 37% for 30 seconds, followed by thorough washing for 20 seconds and the application of the adhesive system without being light-cured.

In the dental substrate, a total etching technique was carried out using 37% phosphoric acid for 30 seconds. The acid was subsequently removed with water before the total drying of the enamel surface and the application of a two-step adhesive system (Case 1: Ambar, FGM; Case 2: Excite, Ivoclar Vivadent) (Figure 15). The surface was gently air-dried to further remove the solvent and adhesive layer was unpolymerized.

**Figure 16:**
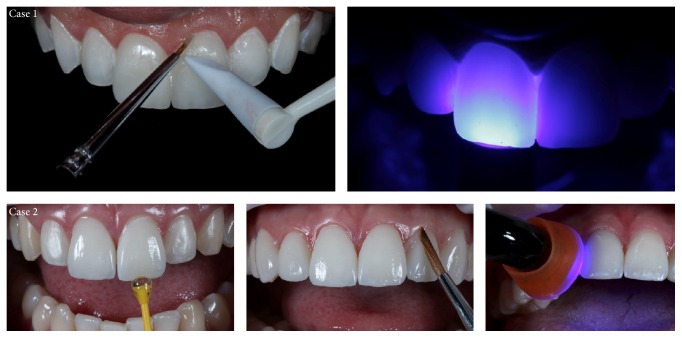
Removal of excess cement with a brush, followed by curing for 90 seconds on each side of the tooth.

The luting agents used in these cases (Case 1: Trans, Allcem Veneer, FGM; Case 2: MV 0, Variolink Veneer, Ivoclar Vivadent) were applied in the internal surface of the veneer, and then the veneer was positioned with light and continuous digital pressure. It is important for cement extravasation to occur on all sides so that the entire inner surface is filled. The excess cement was removed with a brush, and the veneer was light-cured for 10 seconds. Resin cement residues were removed with manual tools and the veneer was once more light-cured at the facial and lingual sides for 90 seconds. (LED Bluephase, Ivoclar Vivadent) (Figure 16).

## 9. Finishing and Polishing

**Figure 17:**
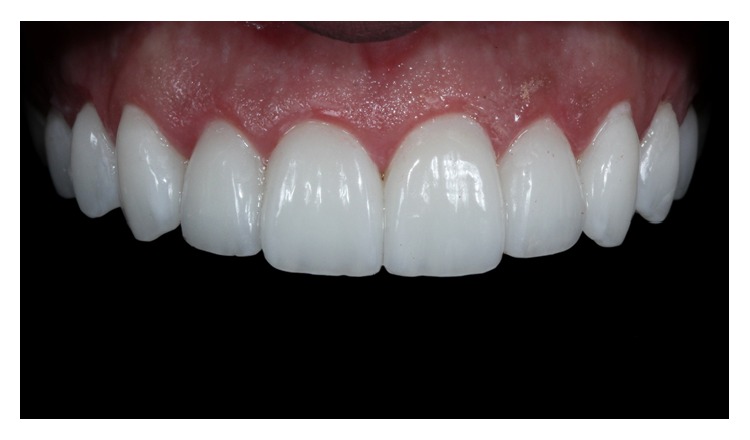
Case 1 immediately after cementation of the ceramic laminates.

**Figure 18 fig18:**
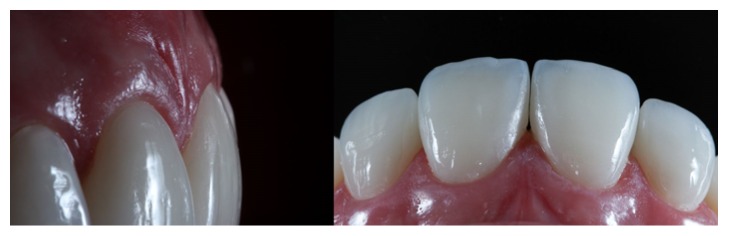
Case 2 immediately after cementation of the ceramic laminates. Ceramic-end-tooth transition with the resin cement, demonstrating periodontal health because the preparations are located at the supragingival level.

**Figure 19 fig19:**
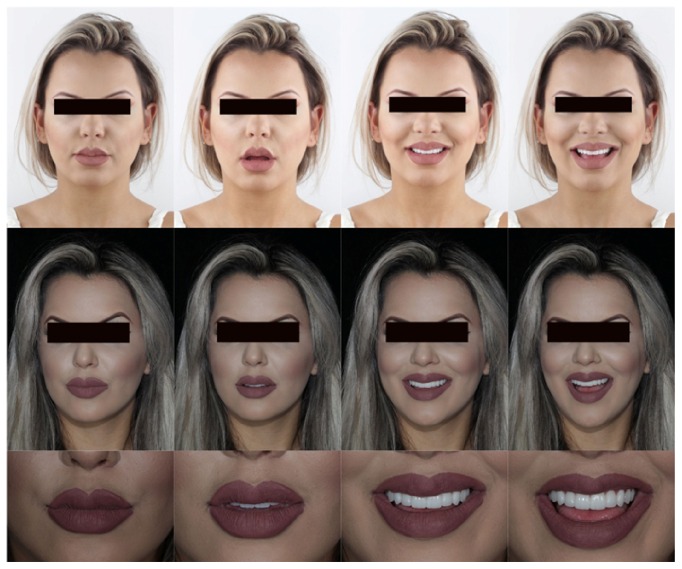
Case 1. Comparison between the initial planning and the finished case. The professional team obtained the patient's desired result by reestablishing dentofacial harmony.

**Figure 20:**
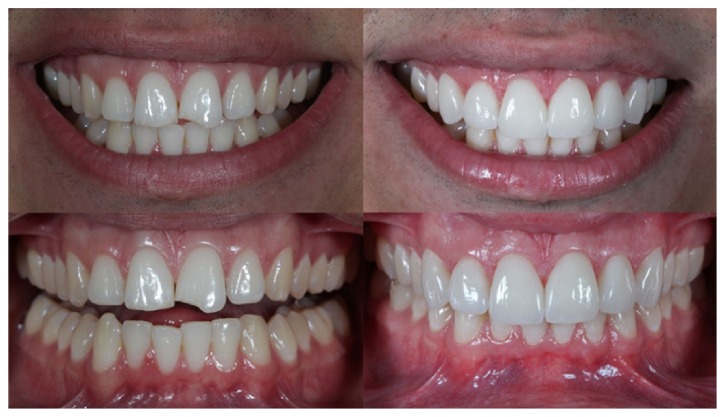
Case 2. The dentolabial and maxillomandibular relationships, before and after treatment.

The finishing and polishing of the cement line were performed with flexible aluminum oxide disks (Sof-Lex XT Pop-On, 3M ESPE, St. Paul, MN, USA). The laminate ceramic contact lens is a restoration that requires no finishing after cementation—only polishing with a scalpel and abrasive rubbers to remove excess cement. That is, the surface of the restoration is not changed by a glaze performed in a laboratory, thus ensuring the stability of the restoration over its years in the patient's mouth. The occlusion was assessed to make sure the anterior guidance and the lateral excursions were correct, while obtaining even occlusal contacts throughout the restorations. Finally, the patients were pleased with the aesthetics and function of the restorations. Furthermore, all prosthetic questions (stability, cement color, point of contact, and marginal adaptation), dentofacial harmony, and relationship dentolabial and maxillomandibular were conferred (Figures 17 [Fig fig18] [Fig fig19]–20).

## 10. Discussion

Aesthetic rehabilitation with ceramic laminates is being increasingly used as a way to preserve tooth structure, especially in young patients. The diagnostic wax mock-up allows for individualized planning and a predictable outcome in cases where a certain shape and position are expected. These procedures require a refined knowledge of tooth anatomy and insight into each patient's personality [19]. The manufacture of this type of restoration has become feasible due to the development of adhesion mechanisms for dental structures. Enamel and dentin etching, combined with the use of primers, adhesives, and resin cements, provide security in this restorative procedure [20].

The first step is to assess the need for periodontal plastic surgery, gingival biotype, and obtain dimensions for the biologic width for each tooth involved in the treatment to determine whether an osteotomy would be needed [21]. By understanding the biologic width for each tooth we can prevent future problems with gingival health and restorations overhangs [22]. The periodontal probe is gently positioned into the gingival sulcus with light pressure to determine the probing depth and gingival biotype [21], which in* Case 1* was a thin tissue biotype. Furthermore, a mock-up was used to allow the surgeon to visualize the final gingival margin and guide the incision shape [21].

Another factor to be considered in the planning is the previous orthodontic treatment to minimize the amount of wear of the tooth structure and provide options for dental alignment problems. Aesthetics is often a primary concern among those seeking treatment through short orthodontic treatment and there are several other factors relating to this that need to be borne in mind when treating the adult patient [23]. However, adult patients desire the resolution of their cases quickly. In* Case 1*, the patient would not want to remake orthodontic treatment, because this was done in childhood and looking for immediate results.

The great advantages of consolidating membership in the enamel increase the indications of ceramic restorations with minimal or no preparation [24]. The majority of cases restored with laminate veneers do not require tooth preparation but rather enamel recontouring [25]. Enamel recontouring is guided by three aspects: (1) need to increase volume to the teeth's facial surface, (2) color of the dental substrate, and (3) path of insertion for the ceramic restorations [25]. In* Case 2*, a minimal and calculated enamel proximal recontouring might be necessary to remove undercuts or to minimize the influence of the proximal height of contour (crest of convexity) [25]. Similarly, the treatment of porcelain with hydrofluoric acid and silane to create an adhesive interface serves as the basis for ceramic laminate veneers, thus developing a structural unit [26]. In this study, care was taken to use a conservative preparation of the enamel, using a silicone mock-up to control the amount of tooth wear necessary to provide a suitable ceramic thickness.

The decision regarding the most appropriate material for these types of situations always leads to questions, as dentists often have difficulty choosing between the use of direct composite resins and the production of ceramic laminates. Dental ceramics can both improve teeth's aesthetic appearance and reestablish their strength and function [10, 27]. When comparing ceramic veneers with composite resins, we can see that the ceramics offer substantial improvements in optical behavior, color stability, shape, surface smoothness, and mechanical and physical properties; ceramic veneers emulate the shape of the dental tissue to be replaced or repaired [28].

To achieve better clinical outcomes in the long term and improve the properties of restorative materials, various studies have been conducted. Improvements in the coefficient of thermal expansion and in the size and distribution of particles have led to abrasive ceramic restorations that are more resistant to fracture, giving an improved prognosis and making the ceramic dental restorative material superior to composite resin [29].

The clincher, however, is that ceramics, both feldspathic and lithium disilicate-reinforced, have almost equal resistance after cementation. The explanation for this is the principle of the adhesive bond, which ensures resistance and bond strength through the transfer of one substrate to another. Thus, there is no advantage to using ceramic lithium disilicate-reinforced ceramics, as they will not result in more resilient restorations relative to pure ceramics, such as feldspar or fluorapatite [15]. By using adhesive feldspathic porcelain restorations, we can return the teeth's original strength [30].

Proper selection of luting material is critical to the clinical longevity of ceramic restorations. Using light-cured resin cements is recommended because they have a variety of colors and different degrees of opacity. In addition, the light-cured luting agent has greater color stability than the dual-curing luting agent [31]. Furthermore, the working time and the degree of flow for the dual-cured cements greatly hinder indications. By contrast, the light-cured luting agent provides a thin cementation line, along with high fluidity and excellent flow grade, facilitating the removal of excess cement [32].

The brush technique seems to be the best option for removing excess resin cement in the clinical routine due to this method's good results and technical simplicity; brushing promotes dental and periodontal health and increases the longevity of aesthetic results [33]. In these cases, cervical margin fall veneers were placed 1 mm above the cement enamel junction, to prevent the restorative material from being eliminated. This decision was based on the mechanical properties of the ceramic material, which exhibits a high modulus of elasticity and has almost nonexistent elastic deformation (these are also known as friability characteristics).

The margin placement of the restorations was located at the supragingival or equigingival (even with the tissue) for all restored teeth [34]. This choice was made to obtain periodontal health, as it reduces subgingival preparation, overcontoured plaque buildup, and difficulty of cleaning; it also facilitates the molding work, as it can be performed without a gingival retraction cord. In addition, the cervical end was located in the enamel, guaranteeing greater longevity of adhesion [35, 36], and the aesthetics were not compromised, as the resin cement mimics the ceramic-end-tooth transition.

The available literature shows that ceramic laminates have performed well over the years. Studies show results ranging from 5 to 20 years of clinical outcomes after the procedure [8, 9, 37, 38]. A functional balance and the correct adjustment of the contacts are also essential to avoid problems, especially fractures of the incisal edges; protrusive and lateral guides should be checked for proper system stability [39]. Successful anterior restorations can be achieved when using a detailed treatment plan and when considering both the aesthetic and the functional parameters [39, 40].

## 11. Conclusion

These clinical reports describe the laminate veneers as an excellent option for effective, conservative, and aesthetic treatment. Therefore, all of the treatment sequences are ruled by the same plan, taking into consideration the adhesive systems, ceramics, ceramic etching, light curing, resin cements, and the correct photographic protocol. As a result, the aesthetics and function expected by the patients were achieved. The use of ceramic veneers enabled a conservative and aesthetic successful rehabilitation treatment. Thus, for the clinical longevity of restorations with laminate veneers, it is necessary for professionals to carefully follow all clinical steps.
